# One Health Perspective of *Salmonella* Serovars in South Africa Using Pooled Prevalence: Systematic Review and Meta-Analysis

**DOI:** 10.1155/2022/8952669

**Published:** 2022-04-20

**Authors:** Tsepo Ramatla, Mpho Tawana, ThankGod E. Onyiche, Kgaugelo E. Lekota, Oriel Thekisoe

**Affiliations:** ^1^Unit for Environmental Sciences and Management, North-West University, Potchefstroom 2531, South Africa; ^2^Department of Veterinary Parasitology and Entomology, University of Maiduguri, P.M.B 1069, Maiduguri 600230, Nigeria

## Abstract

*Salmonella* is a bacterium that is commonly associated with food-borne infections and is regarded as one of the most important pathogens in public health. *Salmonella* serovars, particularly Typhimurium and Enteritidis, which are widely distributed globally, mainly result in outbreaks commonly linked to the consumption of animal products. This study is a systematic review and meta-analysis of studies reporting the prevalence of *Salmonella* serovars from one health perspective that included human, environmental, and animal samples in South Africa. PubMed, ScienceDirect, African Journals Online, and Scopus databases were used to conduct extensive searches of articles which were ultimately included or excluded following the Systematic Reviews and Meta-Analysis (PRISMA) guidelines. According to the data obtained in this review, the overall pooled prevalence estimates (PPE) of *Salmonella* serovars detection were 79.6%, 61.6%, 56.5%, and 43.2% for human, environment, animal, and environment/animal samples in South Africa, respectively. The majority of the studies (50%) used the polymerase chain reaction (PCR) technique for the detection of *Salmonella* serovars, followed by culture methods (26.7%), while 20% used serotyping. The PPE for nontyphoidal *Salmonellae* (NTS) was 65.6% and 34.4% for *Salmonella* Typhimurium and *Salmonella* Enteritidis, respectively. Our data further shows that 3 serovars, namely, *Salmonella* Typhimurium, *Salmonella* Enteriditis, and *Salmonella* Hadar, have been isolated from animals, humans, and the environment in South Africa. Our results highlight the ongoing spread of *Salmonella* spp. especially on animals which might end up infecting humans via direct contact with infected animals or eating infected animal products. This calls for deliberate “One Health” epidemiological studies in order to document information on the transmission between humans, animals, and the environment. This will ultimately result in the formulation of a consolidated salmonellosis control policy by the environmental, human, and veterinary health sectors.

## 1. Introduction

*Salmonella* is a serious public health issue that affects both humans and animals [1]. *Salmonella* strains are grouped as typhoidal and nontyphoidal organisms based on their disease distribution dynamics [2]. Nontyphoidal *Salmonella* (NTS) is responsible for the most important public health problem worldwide including South Africa, and accounts for food-borne illnesses with an estimated 94 million cases globally [3, 4]. According to Backhans and Fellström [5], the majority of *Salmonella* cases are caused by *Salmonella* serovar Enteritidis globally, and the major sources are eggs and poultry meat. Salmonellosis is one of the most serious zoonotic illnesses, affecting both humans and animals around the world [6]. In South Africa, *Salmonella* Typhimurium and Enteritidis are the commonly reported serotypes [7].

Many African countries rely on meat production for their livelihoods, with meat from cattle and poultry serving as a major source of protein in subsistence groups [8]. Various *Salmonella* serovars have been isolated from the gastrointestinal tracts of animals such as chickens, horses, ducks, cattle, pigs, goats, and sheep [9–12]. The published literature on human salmonellosis in South Africa is growing [13–15] including detection of the *Salmonella* serovars from the environment [16–18].

Humans, on the other hand, can become infected by coming into contact with live animals or being in an environment contaminated with animal feces and then accidently ingesting pathogens [8, 19]. The systematic reviews and meta-analyses: a step-by-step guide will be used to quantify and summarize the findings of these studies [20]. Few systematic reviews and meta-analyses studies have been performed in sub-Saharan Africa and Africa in the last ten years on invasive nontyphoidal *Salmonella* (iNTS) disease and the prevalence of *Salmonella* [8, 21–23]. Such reviews focused on surveillance of iNTS infections in humans, in food animals and meat, and antibiotic resistance. All this evidence has pointed out the existence, perpetuation, and dominance of three *Salmonella* enterica serovars Enteriditis, Heidelberg, and Typhimurium in different vertebrate hosts (human and animal) and in environmental samples in South Africa. A better understanding of the existing prevalence of *Salmonella* is required to inform the development and implementation of effective preventative methods. Therefore, the current study was carried out to identify the prevalence gap, analyse and summarize the pooled prevalence of *Salmonella* serovars isolated from humans, animals (chickens, ducks, cattle, pigs, goats, horses, and sheep), and the environment from published data in South Africa by carrying out a systematic review and meta-analysis through a review of published articles.

## 2. Materials and Methods

### 2.1. Study Design

**Figure 1:**
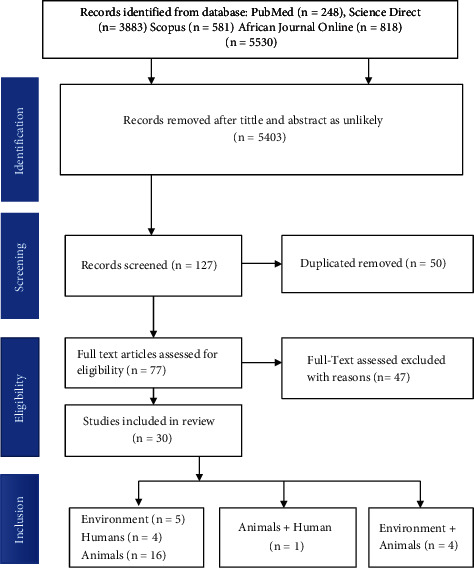
The PRISMA flowchart showing the selection processes of articles on prevalence of *Salmonella* serovars in South Africa.

This study was conducted to estimate the prevalence of *Salmonella* species in South Africa using published articles. The study was conducted in accordance with the Preferred Reporting Items for Systematic Reviews and Meta-Analysis (PRISMA 2020) revised guidelines [24], to report the study (Table S1). The article search approach is presented on a flow chart in Figure 1.

### 2.2. Search Strategy for Relevant Studies

The search was conducted on four databases: PubMed, ScienceDirect, African Journals Online, and Scopus, as described by Ramatla et al. [21]. We searched for all studies published in English from 1980 until June 2021. The keywords that were used in all databases are shown in Table 1. We completed our search on the 24^th^ of June 2021. Titles and abstracts were scanned, and full-text papers were downloaded and accessed from library resources and online platforms. The studies were chosen based on the inclusion and exclusion criteria, which are further listed in the study.

### 2.3. Study and Inclusion Criteria

All available studies and data were included based on the following predefined eligibility criteria. (a) All articles published primarily on quantitative prevalence of *Salmonella* in humans, animals, and environment in South Africa. (b) Articles must clearly state type of samples and methods of diagnosis used, (c) exact numbers of positive samples were clearly stated and lastly, and (d) all published articles were in English language.

### 2.4. Study Exclusion Criteria

Studies were excluded if they were (a) not undertaken in South Africa, (b) book chapters were also excluded, (c) review articles, (d) a smaller sample size (less than 20), (e) articles not reported in English were also discarded, and (f) articles not published between January 1980 and August 2021.

### 2.5. Data Extraction and Data Collection

The titles and abstracts of possible journal articles were scanned and downloaded. To determine eligibility, full versions of possibly relevant articles were obtained. Each article's data, including author names, publication year, location, total number of isolates, and total samples collected, was assembled separately, and entered into a spreadsheet (Microsoft Excel® 2013) and tables were constructed on an MS-word document. The meta-analysis included only journal papers specific to *Salmonella* species or serotype or isolate. If the number of positive *Salmonella* isolates reported exceeds the sample size due to numbers harvested from cultures, the number was recorded at 100% prevalence.

### 2.6. Meta-Analysis

Meta-analysis was performed using Comprehensive Meta-Analysis Version 3.0 (CMA) program (https://www.meta-analysis.com/). The pooled prevalence was calculated using the random-effects model with a 95% confidence interval (CI). Cochran's q statistic and the I-square (I^2^) test were used to measure statistical heterogeneity between studies. The I^2^ scores of more than 75% were regarded to have a high degree of heterogeneity. Heterogeneity with a *P* value less than 0.05 was considered statistically significant. Finally, the funnel plot was generated to investigate the impact of minor research on the pooled *salmonella* prevalence estimated by graphing the prevalence measure against its standard error. The Begg and Mazumdar test was used to determine the asymmetry of the funnel plot.

## 3. Results

### 3.1. Overview of the Selected Studies

This review covered studies from eight provinces of South Africa, namely, Western Cape, Eastern Cape, Northern Cape, North West, KwaZulu-Natal, Gauteng, Limpopo, and Mpumalanga. However, there were no studies reporting on *Salmonella* prevalence in Free State province. All the articles included in this study were published between 1980 and 24^th^ June 2021.

The majority of the studies were carried out from 2010 onwards. All the collected articles were published in English. About 5530 papers were identified through database searching. Articles published primarily on *Salmonella* isolated from humans, animals (chicken, ducks, cattle, pigs, goats, horses, and sheep), and the environment. After reviewing study titles and abstracts, 5403 papers were found to be ineligible. Duplication resulted in the removal of 50 articles. About 77 full articles were assessed for eligibility, and 47 were excluded for the following reasons: studies carried outside of South Africa (*n* = 39), case studies (*n* = 3), and studies without clear total samples collected (*n* = 5). Finally, 30 articles were included in this review and meta-analysis.  Table 2 summarises the studies that were included in this review.

### 3.2. Characteristics of the Eligible Studies That Were Included

From the 30 (Table 2) studies that were included in this study, only 604 samples were from humans, 4111 from animals only (chicken, ducks, cows, pigs, goats, horses, and sheep), 1652 from the environment (water, soil, poultry houses, abattoirs, feed mills, swabs from large animal hospitals, macadamia nuts, manure, and swabs from hospitals), and 180298 from the environment/animal, 69 from the environment/human, and 200 from animals/humans. The number of samples for each study ranged from 22 to 180298. The prevalence amongst the overall studies ranges between 43.2% and 95.8%.

Based on the provinces, the Eastern Cape (*n* = 2749) accounted for most of the samples, followed by KwaZulu-Natal (*n* = 2492), Gauteng (*n* = 1623), the North West (*n* = 691), and lastly by the Western Cape (*n* = 470). However, two studies collected a total of 180320 from all provinces, of which 180298 were from the animals and environment [30], and 22 pediatric wards (humans) [14]. The procedures used to isolate and identify bacterial species from the eligible studies were serotyping, microbiological culture, polymerase chain reaction (PCR), and MALDI-TOF-MS. A total of 10/30 (31.2%) studies used both culture isolation and serotyping of *Salmonella* spp. for identification, 5/30 (15.6%) used the culture method only, while 1/30 (3.3%) studies used a combination of culture isolation, PCR, and MALDI-TOF-MS for *Salmonella* identification, and lastly, 15/30 (50%) studies utilized only PCR for *Salmonella* identification. Fifty-six different *Salmonella* serotypes were identified in this review. We observed that in 13 studies, some *Salmonella* isolates were not identified to the species or serotype level. A total of 999 nontyphoidal *Salmonella* isolates were detected from 14/30 (46.6%) studies, whereas 833/999 (83.4%) were identified as *Salmonella* Typhimurium and 166/999 (16.6%) as *Salmonella* Enteritidis.

### 3.3. Pooling and Heterogeneity of Overall Prevalence of Salmonella Serovars in Animals, Humans, and the Environment

#### 3.3.1. Prevalence Based on Provinces, Study Years, Diagnostic Techniques, Provinces, and Nontyphoidal Salmonellae

**Figure 2:**
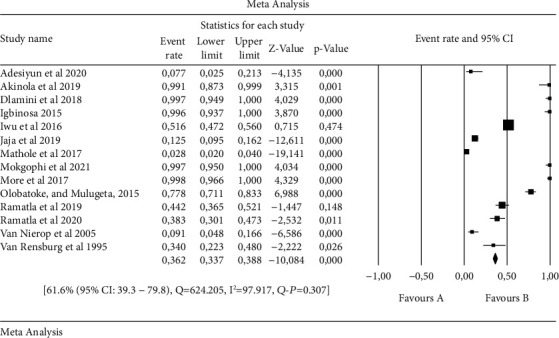
Forest plot showing the pooled estimates of *Salmonella* serovars from animals. The squares demonstrate the individual point estimate. The diamond at the base indicates the pooled estimates from the overall studies.

**Figure 3:**
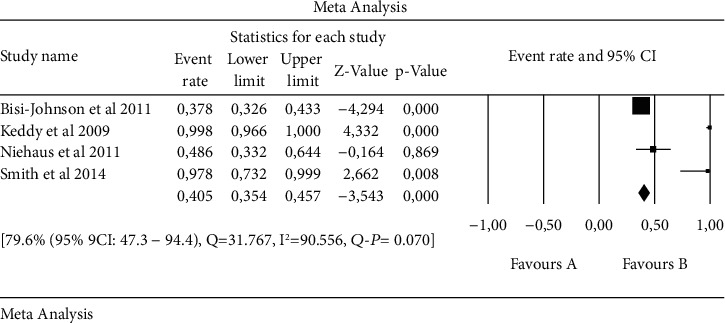
Forest plot showing the pooled estimates of *Salmonella* serovars from human. The squares demonstrate the individual point estimate. The diamond at the base indicates the pooled estimates from the overall studies.

**Figure 4:**
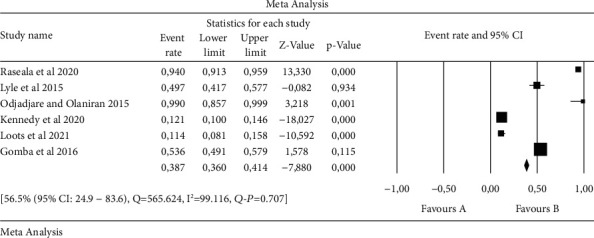
Forest plot showing the pooled estimates of *Salmonella* serovars from environment. The squares demonstrate the individual point estimate. The diamond at the base indicates the pooled estimates from the overall studies.

An overall forest plot showing individual point estimates for the combined prevalence estimates of *Salmonella* serovars in animals, humans, and the environment is presented in Figures 2–4.  Table 3 contains a summary of the subgroup analysis. Significant heterogeneity was seen in humans, animals, the environment, and animal/environment analysis. With regards to the environment/animals, a high degree of heterogeneity was observed [43.2% (95% CI: 11.2–82.1), *Q* = 1003.044, I^2^ = 99.701, *Q-P* = 0.766], followed by the environment with [56.5% (95% CI: 24.9–83.6), *Q* = 565.624, I^2^ = 99.116, *Q-P* = 0.707], then animals with [61.6% (95% CI: 39.3–79.8), *Q* = 624.205, I^2^ = 97.917, *Q-P* = 0.307], while the least observed was in humans with [79.6% (95% 9CI: 47.3–94.4), *Q* = 31.767, I^2^ = 90.556, *Q-P* = 0.070] (Table 3).

High PPE was found from three studies conducted during 2000 − 2010 [95.8% (95% CI: 3.1–10), *Q* = 61.139, I^2^ = 96.729, *Q-P* = 0.351], followed by twenty-six studies conducted during 2010–2021 with a pooled prevalence estimate of 59.7% (95% CI: 40.3–76.4), *Q* = 6364.154, I^2^ = 99.607, *Q-P* = 0.328.

With regards to diagnostic techniques, *Salmonella* serovars were identified using three microbiological diagnostic techniques, whereby serotyping showed the highest prevalence with PPE of 72.1% (95% CI: 34.9–92.5), *Q* = 292,016, I^2^ = 97.945, *Q-P* = 0.237 with ten studies, followed by polymerase chain reaction (PCR) [65.7% (95% CI: 51.1–77.8), *Q* = 729,46, I^2^ = 98.081, *Q-P* = 0.036] with fifteen studies, and a culture-based technique of [41.5% (95% CI: 16.7–71.5), *Q* = 493,370, I^2^ = 98,581, *Q-P* = 0.596] with five studies.

The PPE of *Salmonella* serovars was higher in Western Cape province [98.7% (95% CI: 68.3–100), *Q* = 17,791, I^2^ = 88,759, *Q-P* = 0,017], followed by five studies in the North-West [76.6% (95% CI: 52.1–90.8), *Q* = 79.750, I^2^ = 94,984, *Q-P* = 0,035], eight studies in Gauteng [65.6% (95% CI: 39.0–85.1), *Q* = 273,845, I^2^ = 97,444, *Q-P* = 0.247], six studies in KwaZulu-Natal [42.7% (95% CI: 14.7–76.3), *Q* = 564,052, I^2^ = 99,114, *Q-P* = 0,695]. The province with the lowest PPE was the Eastern Cape with six studies [35.6% (95% CI: 15.8–62.1), *Q* = 454,089, I^2^ = 98,899, *Q-P* = 0.285].

In this review, a total of 1047 NTS isolates were reported. The highest PPE of NTS was observed from S. Typhimurium with 65.6%. S. Enteritidis had the lowest prevalence of 34.4% [(95% CI: 12.7–65.4), *Q* = 75,133, I^2^ = 96,007, *Q-P* = 0.322] based on seven studies. As shown in Table 4, Gauteng was the most dominant province with (*n* = 769) NTS isolates, followed by the North West (*n* = 72), KwaZulu-Natal (*n* = 19), and the Northern Cape (*n* = 6), whilst Limpopo and the Eastern Cape with only one isolate. The study conducted from all nine provinces by Smith et al. [14] reported 46 NTS isolates.

### 3.4. Dominant Serotypes and One Health Perspective

**Figure 5:**
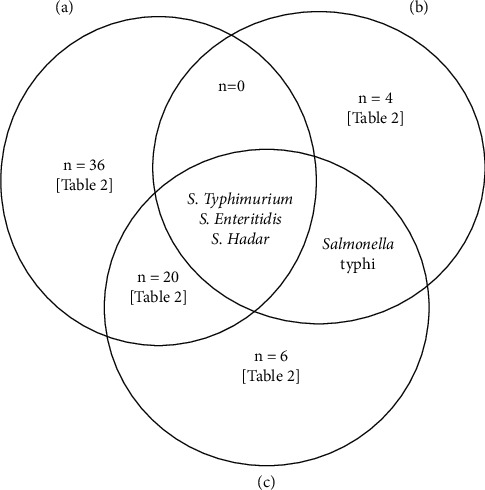
Venn diagram showing the shared isolates between (a) animals, (b) humans, and (c) the environment.

Of the 30 studies, 51 *Salmonella* isolates were identified. These serotypes were found in at least two or more studies: S. Enteritidis, S. Agona, S. Heidelberg, S. Newport, S. *bongori*, S. *enterica*, S. Typhimurium, S. pullorum, S. Hadar, S. Schwarzengrund, S. anatum, S. Seftenberg, S. Montevideo, S. Mbandaka, S. Cardoner, and S. Hayindongo. From 15 studies, some *Salmonella* isolates were not typed.  Figure 5 shows the presence of S. Typhimurium, S. Enteritidis, and S. Hadar in both animals, humans, and environment. In addition, S. Heidelberg S. Newport, and S. Agona were also detected from both the animals and environmental samples. All the isolates from human, animals, and environment are listed in Table 2.

### 3.5. Publication Bias

**Figure 6:**
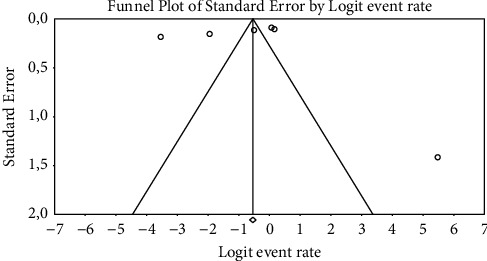
Funnel plot with 95% confidence limits of the pooled prevalence of the studies conducted in Eastern Cape province.

The Begg and Mazumdar rank correlation test demonstrated no significant publishing bias for practically all parameters except for the studies conducted in Eastern Cape province, where both the asymmetry of the funnel plots and the *P* value of 0.045 indicated considerable bias (Table 3 and Figure 6).

## 4. Discussions

The data obtained from 30 published studies showed an overall prevalence of 66.3% for *Salmonella* serovars in South Africa which is higher than the findings of the studies conducted in Africa, the Middle East, North Africa, and Africa which reported the prevalence of *Salmonella* serovars at 5.7%, 8.8%, and 44.8%, respectively [20, 45, 46]. The PPE of *Salmonella* serovars was high in humans with 79.6%, animals with 61.6%, the environment with 56.5%, and the environment/animal with 43.2%. This is higher compared to a similar systematic review and meta-analysis conducted in Iran with 6.89% in animals [47], (6.6%) in humans in the Middle East and North Africa (MENA) [20], and human (8.4%) in sub‐Saharan Africa [48]. The difference could be explained by microbiological diagnostic procedures employed, antibiotic resistance prevention and control practices, and differences in *Salmonella* serovars isolated. Contamination of food products and close contact with livestock animals could be a source of *Salmonella* transmission to humans [49].

Nontyphoidal *Salmonella* (NTS) infections induce gastroenteritis in people because the bacteria cause an invasive, extra-intestinal condition that leads to bacteraemia and localized systemic infections known as invasive NTS [50]. In this study, nine hundred and forty-one (941) serovars of S. Enteritidis and S. Typhimurium were identified as NTS. They were also identified as the most prevalent serotypes with Gauteng province having the highest number of NTS isolates. There were about 4.9% of NTS identified from the study conducted by Smith et al. [14] from all nine provinces. According to the systematic review conducted in the Middle East and North Africa, *Salmonella* Typhimurium and Enteritidis were the most prevalent serotypes [20]. The S. Typhimurium and S. Enteritidis are among the top five most common serotypes reported in the United States [51], and they are associated with salmonellosis [52–54]. The current study recorded NTS *Salmonella* from both humans and the environment, and animals dominated by 86.8% of S. Typhimurium, followed by 13.2% of S. Enteritidis. Our study showed that the prevalence of NTS was higher in animal samples. By contrast with the findings in America [55], reported a high prevalence of NTS from the environmental samples. These findings quantify the existing NTS status in animal production while also emphasizing the main public health hazard associated with the presence of NTS in the animal production chain, which may eventually affect humans.

Traditional microbiological methods such as culture isolation using the spread plate technique are still considered the gold standard for diagnostic tests since they efficiently allow the identification of different bacterial species [56]. Generally, the International Organization for Standardization (ISO-6579, 2002) recommends classical microbiological culture isolation for the identification of *Salmonella* spp. (ISO, 2002). About 41.5% of the studies included in this review used culture-based methods. The culturing method is time-consuming, labour-intensive, and sometimes has low sensitivity which makes it unsuitable for regular examination of large numbers of samples as opined by different researchers [11, 12, 17, 28, 37, 57]. Due to its low sensitivity, some studies combine the culturing method with other sensitive molecular techniques such as PCR [58]. The combination of traditional culture isolation and molecular methods accurately identify bacterial pathogens [58]. Moreover, research has also shown that using molecular methods (PCR) reveals more identification of bacterial pathogens than traditional culture-based methods [56, 58]. In this study, we observed that PCR appears to be the most utilized diagnostic method in the detection of *Salmonella* serovars/isolates with a prevalence of 72.1%, as employed in about 15 studies with over 4219 samples screened. However, research should also not exclude one or the other technique, as a polyphasic approach has proven ideal to investigate bacterial pathogens in microbiological practice [59]. All the studies included in this study were carried out in diverse ways, with some examining a high number of samples and others using various diagnostic approaches. The data on publication bias analysis leads us to conclude that numerous factors, including sample size and different diagnostic approaches could be responsible for the substantial heterogeneity between the findings.

This systematic review and meta-analysis comprised studies from different provinces, of which most of the studies (*n* = 26, 86.7%) were conducted between 2010 and 2021. Then followed by the period of the year 2000 to 2010 with 10% of the studies which screened 187547. From year period 1980–1990 and 1990–2000, PPE were not calculated because there was only one eligible study for each period with 501 samples screened [9, 41]. This low number of studies might be due to lack of facilities and funds for conducting research at that time or salmonellosis was a neglected disease in that period in South Africa. In terms of diagnostic methods, these two studies employed serotyping for detecting *Salmonella* serovars/isolates. The Free State province was not represented in the data sets due to unavailable published data on the prevalence of *Salmonella* spp. Gauteng appeared to be a province with high number of published articles which may be connected with the availability of resources to conduct research.

Our analysis indicates an increase in the number of samples tested from 1980 to 2021. These findings could be attributed to higher consciousness by researchers and the health sector about zoonotic diseases which included *Salmonella* serovars as well as available advanced equipment to conduct research as the majority of recent studies (2010–2021) used molecular methods such as PCR, as compared to the studies of the period 1980 to 2000. Therefore, health officials/authorities should be concerned about the rising incidence of these bacteria.

According to the World Health Organization (WHO), food safety, zoonotic disease control, laboratory services, neglected tropical diseases, environmental health, and antimicrobial resistance are among the areas of work where a “One Health” approach is particularly pertinent (https://www.euro.who.int/en/home). Therefore, the presence of zoonotic isolates, namely, S. Typhimurium, S. Enteritidis, and S. Hadar in both animals and humans, and the environment should be taken into consideration in South Africa. The two most commonly reported serotypes of non-typhoidal Salmonella are S. Typhimurium and S. Enteritidis. NTS is a common cause of invasive bacterial disease and is linked to death. Transmission of *Salmonella* isolates includes animals, animal products, water, and infected humans [14]. Furthermore, S. Heidelberg, S. Newport, and S. Agona were also detected from both the animals and the environmental samples. This indicates that there is the possibility of ongoing circulation of the serovars.

### 4.1. Significance/Strengths of the Study

This study has a considerable number of strengths including: (1) the present meta-analysis provides the estimates for the prevalence of *Salmonella* on animals, human, and the environment in South Africa and revealed that there are some provinces with few/no studies conducted. (2) This study selected high-quality peer-reviewed studies to give an overview of unbiased data on the prevalence of *Salmonella* species isolated from humans, the environment, and animals in South Africa. (3) There are no human studies published in the North West, Limpopo, Gauteng, Mpumalanga, and Northern Cape provinces. (4) Additionally, this study demonstrated that *Salmonella* serovars infect both humans and animals and have been detected in the environmental samples. This observation suggests the need for a consolidated “One Health” approach from the ecological, human, and animal health sectors.

## 5. Limitations of the Study

Several limitations have been identified which include the following: (1) the majority of the studies included samples between 1980 and 2021; (2) the number of articles from some provinces (Western Cape, Eastern Cape, Northern Cape, North West, KwaZulu-Natal, Gauteng, Limpopo, and Mpumalanga) was unusually high, which may have influenced the overall estimate, whilst provinces such as Free State, Limpopo, Mpumalanga, and Northern Cape were underrepresented; (3) some studies reported the total number of isolates from cultures without showing the number of individual serotypes; (4) the studies included in our analyses showed a high level of heterogeneity, hence readers should exercise caution when interpreting the pooled analysis and subgroup; (5) our bias assessment revealed the overall risk of bias from sample size and different diagnostic methods used and sample selection; (6) few reports from humans were observed whereby only four studies were undertaken in Gauteng, Eastern Cape, KwaZulu-Natal, and from all the provinces; and (7) PPE of *Salmonella* in animal/human, and environment/human were not calculated because there are single reports on each.

## 6. Conclusion

Our systematic review and meta-analysis showed the prevalence of *Salmonella* serovars from animals, humans, and the environment in South Africa. This study highlights the huge knowledge gap on salmonellosis in this country. There are significant gaps in surveillance and a lack of published studies on the prevalence of *Salmonella* spp. in some provinces like Free State province. The results demonstrated that a high prevalence of *Salmonella* serovars is noticeable in animals rather than in humans and the environment. These emphasize the main public health hazard associated with the presence of *Salmonella* serovars, especially NTS in the animal production chain, which may eventually affect humans. Several studies had methodological data gaps, which cast doubt on their validity and made comparisons difficult. The fact that *Salmonella* serovars infect both humans and animals and have been detected in environmental samples means there is a need for a consolidated “One Health” approach from the ecological, human, and animal health sectors in terms of epidemiological, therapeutic, and policy formulation research.

## Figures and Tables

**Table 1:** Databases and search string.

No.	Source	Search string	Results
1	PubMed (https://www.ncbi.nlm.nih.gov)	Prevalence AND *Salmonella* species OR diagnosis OR salmonellosis OR human OR animal OR environment AND South Africa	248
2	ScienceDirect (https://www.sciencedirect.com)	Prevalence AND *Salmonella* species OR diagnosis OR salmonellosis OR human OR animal OR environment AND South Africa	3 883
3	Scopus (https://www.scopus.com)	Prevalence AND *Salmonella* species OR diagnosis OR salmonellosis OR human OR animal OR environment AND South Africa	581
4	AJOL (https://www.ajol.info/index.php/ajol)	Prevalence AND *Salmonella* species OR diagnosis OR salmonellosis OR human OR animal OR environment AND South Africa	818

**Table 2:** Overview of the numbers of research article on *Salmonella* serovars that were included in this systematic review and meta-analysis.

Study (citation)	Province	Diagnosis method	Sample size	Isolates: (prevalence%)	Study population	*Salmonella* serovar
[15]	KwaZulu-Natal	Culture	37	18: (49%)	Human	S. Enteritidis
[25]	Western Cape	Culture	172	172: (100%)	Animal/environment	*Salmonella* spp.
[12]	Gauteng	PCR	151	151: (100%)	Animal	S. Hadar, S. Dublin, S. Enteritidis, S. Mbandaka, S. Saintpaul, S. Thompson, S. infantis, and S. Agona
[26]	Gauteng	Culture	147	73: (50%)	Environment	S. Heidelberg, S. Kibusi, S. Kottbus, S. Orion, S. Typhimurium, and S. Virchow
[27]	Gauteng	MALDI-TOF-MS	491	263: (54%)	Environmental	S. Muenchen, S. Typhimurium, S. Heidelberg, S. Bsilla
[28]	North West	PCR	274	114: (42%)	Animal	S. Typhimurium, S. Enteritidis, S. Newport, S. Heidelberg, S. Bongori, S. enterica serovar Paratyphi B, S. Tennessee, and S. Pullorum
[29]	Gauteng	Culture	39	3: (8%)	Animal	*Salmonella* spp.
^*∗*^[30]	South Africa	Culture	180298	9031: (5%)	Animals/environment	S. Seftenberg, S. Montevideo, S. Ohio, S. Muechen, S. Schwarzengrund, S. Anatum, S. Mbandaka, S. Hadar, S. Infanits, and S. Orion
[18]	Mpumalanga	Culture	264	36: (14%)	Environment	*Salmonella* spp.
[13]	Eastern Cape	PCR	315	119: (38%)	Human	S. Choleraesuis, S. Enteritidis, S. Eppendorf, S. Hadar, S. Isangi, S. Panama, S. typhi, S. Typhimurium, and untyped *Salmonella*
[31]	Eastern Cape	PCR	384	48: (13%)	Animal	S. Enterica
[16]	Eastern Cape and KwaZulu-Natal	PCR	361	195: (54%)	Environment	*Salmonella* spp.
[32]	North West	PCR	32	32: (100%)	Animal	*Salmonella* spp.
[33]	Eastern Cape	PCR	500	258: (52%)	Animal	*Salmonella* spp.
^*∗*^[14]	South Africa	Serotyping	22	22: (100%)	Human	*Salmonella* Typhimurium
[11]	North West	PCR	55	55: (100%)	Animal	S*. bongori*, S. Pullorum, S. Typhimurium, S. Weltevreden, S. Chingola, S. Houten, and S. Bareily
[34]	Limpopo, Eastern Cape, Northern Cape, North West and KwaZulu-Natal	Serotyping	1069	30: (3%)	Animal	S. Chester, S. Cardoner, S. Sambrae, S. Typhimurium, S. Schwarzengrund, S. A. Århus, S. Pomona, S. Senftenberg, and S. Techimani
[35]	Eastern Cape	PCR	120	120: (100%)	Animal	*Salmonella* spp.
[36]	KwaZulu-Natal	PCR	48	48: (100%)	Environment	*Salmonella* spp.
[37]	KwaZulu-Natal	PCR	200	146: (73)	Animal	*Salmonella* spp.
[38]	Limpopo	PCR	604	92: (15%)	Animal/environment	S. Heidelberg, S. Aberdeen, S. Hayindongo, S. Mbandaka, S. Anatum, S. Othmarschen, S. Nigeria, S. Tennessee, S. Cardoner, S. Senftenberg, and S. Pretoria
[39]	Western Cape	Culture	229	229: (100%)	Animal	*Salmonella* spp.
[17]	Gauteng	PCR	416	391: (65)	Environment	S. Heidelberg, S. Enteritidis, S. Newport, S. Agona, S. Typhimurium, and S. Montevideo
[40]	KwaZulu-Natal	PCR	777	94: (12)	Environment	*Salmonella* spp.
[9]	Gauteng	Serotyping	50	17: (34%)	Animal	S. Hayindogo, S. Typhimurium, S. Agona, S. Kingston, S. Braenderup, S. Mbandaka, and S. Istanbul
[41]	Western Cape	Serotyping	69	57: (83%)	Human/environment	*Salmonella typhi*
[10]	North West	Serotyping	150	150: (100%)	Animal	*Salmonella* spp.
[42]	Gauteng	Serotyping	230	230: (100%)	Human	S. Typhimurium
[43]	North West	PCR	180	140: (78%)	Animal	S. Typhimurium, S. Enteritidis, and S. Newport
[44]	Gauteng	Culture	99	9: (9%)	Animal	S. Hadar, S. Heidelberg, S. Derby, S. Typhimurium, S. Westhampton, S. Schwarzengrund, S. Virchow, S. Reading, S. Anatum, S. Irumu, and S. Blockley

^*∗*^Article does not specify sampled provinces. PCR = polymerase chain reaction; MALDI-TOF-MS = matrix assisted laser desorption ionization-time of flight mass spectrometry.

**Table 3:** Proportion of *Salmonella* serovars isolated from humans, the environment and animals, screening methods, study year, and sampling sites.

Risk factors	Number of studies	Pooled estimates			Measure of heterogeneity			Publication bias
		Sample size	Number of positive	I^2^ (95%CI)	Cochran's Q	Heterogeneity I^2^ (%)	*Q-P*	Begg and Mazumdar rank *P*value
*Overall study*								
Human	4	604	399	79.6 (47.3–94.4)	31.767	90.556	0.070	0.08712
Environment	6	1652	636	56.5 (24.9–83.6)	565.624	99.116	0.707	0.28651
Animal	14	4111	1733	61.6 (39.3–79.8)	624.205	97.917	0.307	0.39215
Animal/human	1	200	146	—	—	—	—	—
Environment/animal	4	181435	9489	43.2 (11.2–82.1)	1003.044	99.701	0.766	0.50000
Environment/human	1	69	57	—	—	—	—	—

*Study year*								
1980–1990	1	69	57	—	—	—	—	—
1990–2000	1	50	17	—	—	—	—	—
2000–2010	3	501	681	95.8 (3.1–10)	61.139	96.729	0.351	0.30075
2010-2021	26	187549	11461	59.7 (40.3–76.4)	6364.154	99.607	0.328	0.00335

*Diagnostic technique*								
PCR	15	4219	2245	65.7 (51.1–77.8)	729.466	98.081	0.036	0.09075
Culture	8	181285	9841	41.5 (16.7–71.5)	493.370	98.581	0.596	0.50000
Serotyping	6	2367	1182	72.1 (34.9–92.5)	292.016	97.945	0.237	0.22634
MALDI-TOF-MS	1	491	39	—	—	—	—	—

*Nontyphoidal Salmonella*								
S. Typhimurium	13	817	885	65.6 (34.6–87.3)	75.133	96.007	0.322	0.50000
S. Enteritidis	7	124	166	(12.7–65.4)	75.133	96.007	0.322	0.50000

*Provinces*								
KwaZulu-Natal	6	2492	683	42.7 (14.7–76.3)	564.052	99.114	0.695	0.42549
Gauteng	8	1623	1452	65.6 (39.0–85.1)	273.845	97.444	0.247	0.50000
Eastern Cape	6	2749	800	35.6 (15.8–62.1)	454.089	98.899	0.285	0.04544
North-West	5	691	714	76.6 (52.1–90.8)	79.750	94.984	0.035	0.50000
Northern Cape	1	1069	30	—	—	—	—	—
Mpumalanga	1	264	36	—	—	—	—	—
Limpopo	2	1333	122	—	—	—	—	—
Western Cape	3	470	158	98.7 (68.3–100)	17.791	88.759	0.017	0.30075

PCR = polymerase chain reaction; MALDI-TOF-MS = matrix assisted laser desorption ionization-time of flight mass spectrometry.

**Table 4:** Published articles of occurrence of nontyphoidal *Salmonella* from human, environment, and animals.

Province	NTS isolates	S. Typhimurium	S. Enteritidis	Studies
KwaZulu-Natal	19	1: (5%)	18: (95%)	[15, 34]
Gauteng	796	710: (89%)	86: (11%)	[9, 12, 17, 26, 42, 44]
Eastern Cape	1	1: (100%)	−	[34]
North West	72	53: (74%)	19: (26%)	[11, 28, 34, 43]
Northern Cape	6	5: (83%)	1: (17%)	[13, 34]
Mpumalanga	—	—	—	—
Limpopo	1	1: (100%)	—	[34]
Western Cape	—	—	—	—

NTS = nontyphoidal *Salmonella*.

## Data Availability

The datasets generated and analysed will be available on request to the corresponding author.

## Supplementary Materials

Supplementary Materials Table S1. Checklist of items to include when reporting a systematic review or meta-analysis.
